# Serum ****β****-Catenin Levels Associated with the Ratio of RANKL/OPG in Patients with Postmenopausal Osteoporosis

**DOI:** 10.1155/2013/534352

**Published:** 2013-04-22

**Authors:** Xiao-Juan Xu, Lin Shen, Yan-Ping Yang, Rui Zhu, Bo Shuai, Cheng-Gang Li, Man-Xiang Wu

**Affiliations:** Department of Integrated Traditional Chinese Medicine and Western Medicine, Union Hospital, Tongji Medical College, Huazhong University of Science and Technology, Wuhan, Hubei 430022, China

## Abstract

*Objective*. To demonstrate the role of Wnt/**β**-catenin canonical pathway in postmenopausal osteoporosis by evaluating serum **β**-catenin levels in patients with postmenopausal osteoporosis and analyzing their possible relationship with serum OPG, RANKL, the ratio of RANKL/OPG, sclerostin, and bone turnover markers. *Methods*. 480 patients with postmenopausal osteoporosis and 170 healthy postmenopausal women were enrolled in the study. Serum **β**-catenin, OPG, RANKL, and sclerostin levels were measured by enzyme-linked immunosorbent assay. Bone status was assessed by measuring bone mineral density and bone turnover markers. Estradiol levels were also detected. *Results*. Serum **β**-catenin levels were lower in postmenopausal osteoporotic women compared to nonosteoporotic postmenopausal women (26.26 ± 14.81 versus 39.33 ± 5.47 pg/mL, *P* < 0.001). Serum **β**-catenin was positively correlated with osteoprotegerin (*r* = 0.232, *P* < 0.001) and negatively correlated with the ratio of RANKL/OPG, body mass index, and sclerostin (*r* = −0.128, *P* = 0.005; *r* = −0.117, *P* = 0.010; *r* = −0.400, *P* < 0.001, resp.) in patients with postmenopausal osteoporosis. *Conclusion*. The results indicate that lower serum **β**-catenin and concomitantly higher ratio of RANKL/OPG may be involved in the pathogenesis of postmenopausal osteoporosis. Functional communication between RANKL/RANK/OPG system and Wnt pathways plays an important role in postmenopausal osteoporosis.

## 1. Introduction 

Postmenopausal osteoporosis (PMOP) is a highly prevalent disease, characterized by reduced bone mass, leading to increased bone fragility and fracture risk, caused by estrogen deficiency. A lot of recent reports provide evidence that the Wnt/*β*-catenin (canonical) signaling, one of the three known pathways of Wnt signaling, may be implicated in pathogenesis of postmenopausal osteoporosis. The Wnt/*β*-catenin pathway is essential for normal osteogenesis [1–3]. The Wnt/*β*-catenin canonical pathway is modulated by a number of factors that include Dickkopf (Dkk-1) and sclerostin, which compete with the Wnt/*β*-catenin for binding to LRP5/6, disrupting (Dkk-1) or antagonizing (sclerostin) LRP5/6 mediated Wnt signaling [4]. Receptor activator of NF-*κ*B ligand (RANKL) is highly expressed on the surface of bone marrow stromal cells (BMSCs) and preosteoblasts [5]. When RANKL binds to RANK, osteoclast differentiation and function are enhanced [6]. Osteoprotegerin (OPG), produced by BMSCs and osteoblasts, is a soluble decoy receptor to inhibit RANK-RANKL-mediated osteoclastogenesis [7, 8]. de Toni et al. reported that OPG expression is regulated by *β*-catenin in colon cancer cells [9]. Recent evidence implicated that sclerostin, a major Wnt/*β*-catenin antagonist, stimulates expression of RANKL [10, 11]. Based on recent studies, we hypothesize that some relationships may exist between Wnt/*β*-catenin signaling and RANKL/RANK/OPG system in PMOP.

To demonstrate the role of Wnt/*β*-catenin canonical pathway in postmenopausal osteoporosis, we evaluated the levels of serum *β*-catenin, OPG, RANKL, sclerostin, and bone turnover markers in postmenopausal osteoporotic patients and compared them to those in postmenopausal nonosteoporotic women. In addition, we analyzed the relationships of *β*-catenin with OPG, RANKL, the ratio of RANKL/OPG, sclerostin, and bone turnover markers.

## 2. Subjects and Methods

### 2.1. Study Population

Our cross-sectional study included 480 postmenopausal women with osteoporosis and 170 healthy postmenopausal women as a control group. According to the principle of statistics, the PMOP patients were enrolled from 4 hospitals (2 in Hubei province, 1 in Jiangxi province, and 1 in Jilin province in China) by advertisement recruiting during the period of December 2009 and March 2012 in China. Eligible PMOP participants were required to have a natural menopausal history of 2–10 years and a BMD T-score of <−2.5 at the lumbar spine using dual-energy X-ray absorptiometry (DXA). Exclusion criteria were as follows: (1) treatment with calcitonin, bisphosphonates, raloxifene, estrogen, or estrogen/progestogens within 12 months, (2) coexistence of any other metabolic bone disease except for osteoporosis, (3) severe chronic disease, including malignancy, (4) medication that could affect bone metabolism, (5) previous radiation therapy, and (6) abnormal liver and kidney function tests. Healthy postmenopausal women were enrolled from physical examination center in Union Hospital, Tongji Medical College, Huazhong University of Science and Technology. After a medical examination, they were excluded from osteoporosis and other diseases affecting bone metabolism. The protocol was approved by the responsible Clinical Trial Ethics Committee. Written informed consent was obtained from each participant.

### 2.2. Clinical Evaluation

In all subjects, we measured height and weight and calculated body mass index (BMI) using the Quetelet formula (weight in kilograms divided by the square of height in meters). Bone mineral density (BMD) was measured for the anteroposterior lumbar spine (L1–L4) by dual-energy X-ray absorptiometry (DXA) (Lunar Prodigy Advance; GE Healthcare, Madison, WI, USA). A control phantom was scanned every day, and all DXA measurements were performed by experienced operators in every hospital. Osteoporosis was defined as a T-score of <−2.5 at the lumbar spine.

### 2.3. Laboratory Data

Venous blood samples were taken in the morning between 8:00 AM and 9:00 AM after an overnight fast. The samples were centrifuged for 10 minutes at approximately 3000 r/min within 30 minutes, and the serum was separated and stored at −80°C prior to analysis. 

Serum *β*-catenin, RANKL, OPG, and sclerostin levels were measured by enzyme-linked immunosorbent assay (ELISA, Yanhui biotechnology Co., Ltd., Shanghai, China). According to the manufacturer's instructions, the minimum detectable amount of human *β*-catenin and OPG kit was less than 1.0 pg/mL. The minimum detectable amount of human sclerostin and RANKL kit was less than 1.0 pmol/L. Intra- and interassay coefficients of variation were less than 15%. No significant cross-reactivity or interference was observed. Serum *β*-isomerized C-terminal crosslinking of type I collagen (CTX), intact N-terminal propeptide of type I collagen (PINP), N-mid fragment of osteocalcin (N-MID-OT), and 25-hydroxyvitamin D (25(OH)D) levels were measured using automated Roche electrochemiluminescence system. Serum estradiol levels were measured by electrochemiluminescence system in the Department of Nuclear Medicine. Intra- and interassay variations were <6% in our laboratory.

### 2.4. Statistical Analysis

All data for continuous variables were described as mean ± SD. Serum levels of *β*-catenin and other parameters between patients and controls were compared by independent-samples *t*-test. Spearman's coefficient of correlation was used for correlation between serum levels of *β*-catenin and other parameters in both groups. Multiple regression analysis was used to determine the influence of one independent variable after correcting for others. All statistics were analyzed using SPSS 16.0 software. A *P* value of less than 0.05 was considered statistically significant in all tests.

## 3. Results

The main characteristics and laboratory data of the study population were listed in Table 1. There was no statistically significant difference between patients and controls for age, weight, height, and BMI. CTX and PINP serum concentrations were higher in postmenopausal osteoporotic women than in nonosteoporotic postmenopausal women. There was no significant difference between patients and controls for serum estradiol levels, N-MID-OT levels, 25(OH)D levels, and OPG levels. The differences for *β*-catenin, sclerostin, and RANKL levels between two groups were significant, with *P* < 0.001.

**Figure 1:**
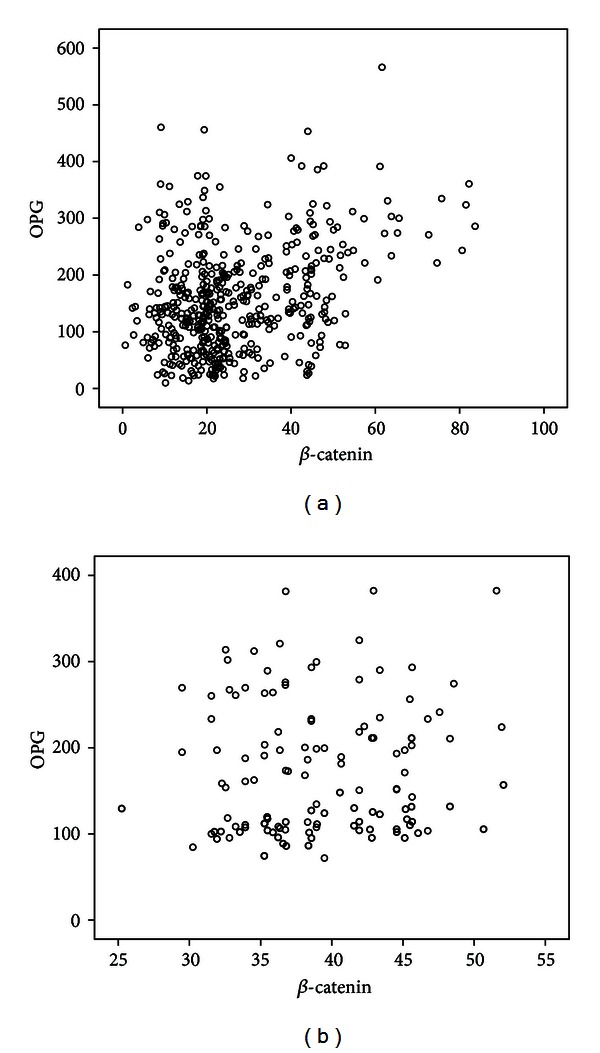
Univariate correlation (Spearman analysis) between *β*-catenin and OPG serum levels in PMOP patients ((a) *r* = 0.232, *P* < 0.001) and controls ((b) *r* = 0.112, *P* = 0.145).

**Figure 2:**
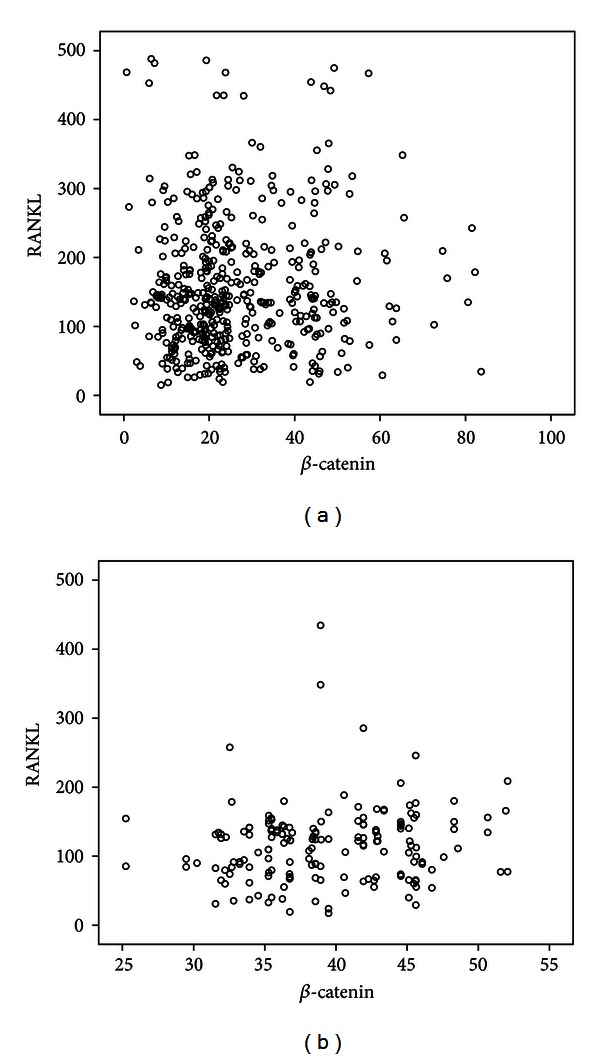
Univariate correlation (Spearman analysis) between *β*-catenin and RANKL serum levels in PMOP patients ((a) *r* = 0.067, *P* = 0.143) and controls ((b) *r* = 0.134, *P* = 0.081).

**Figure 3:**
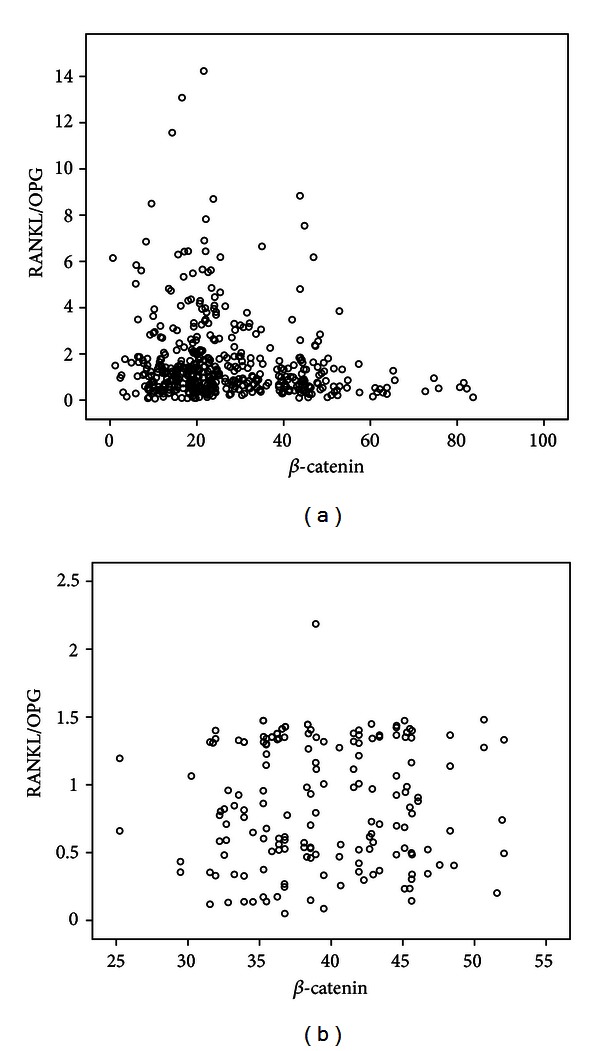
Univariate correlation (Spearman analysis) between *β*-catenin and RANKL/OPG serum levels in PMOP patients ((a) *r* = −0.128, *P* = 0.005) and controls ((b) *r* = 0.032, *P* = 0.674).

**Figure 4:**
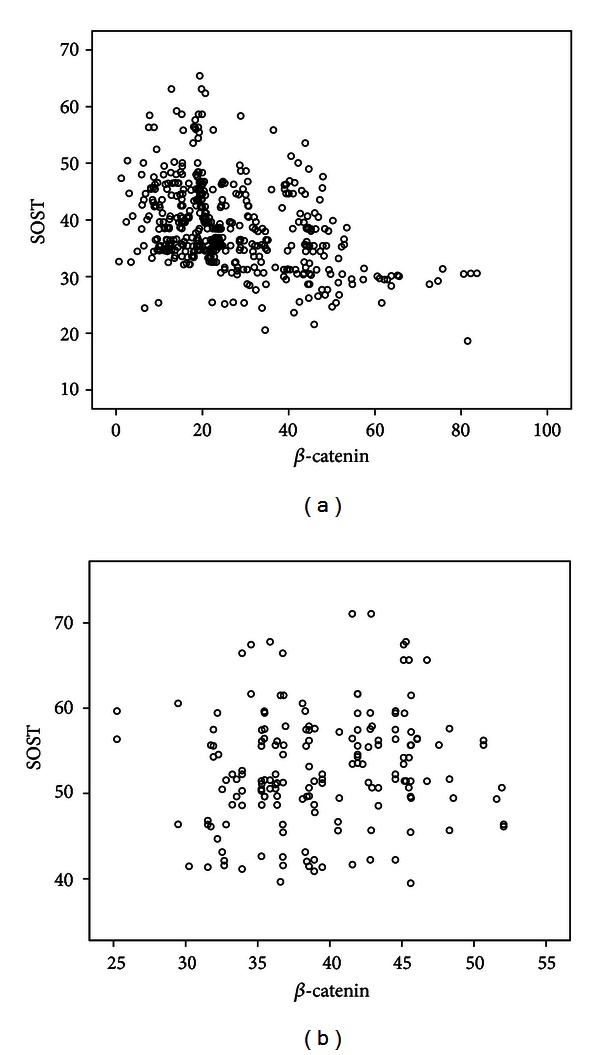
Univariate correlation (Spearman analysis) between *β*-catenin and sclerostin serum levels in PMOP patients ((a) *r* = −0.400, *P* < 0.001) and controls ((b) *r* = 0.145, *P* = 0.060).

We examined the correlations between serum *β*-catenin levels and various parameters in patients and controls. As shown in Table 2, serum *β*-catenin levels were positively correlated with OPG and negatively correlated with sclerostin, the ratio of RANKL/OPG, BMI, and BMD. No correlation between serum *β*-catenin and age, estradiol, N-MID-OT, 25(OH)D, CTX, PINP, and RANKL was found. No correlation between serum *β*-catenin and other parameters was observed in control group. A multiple regression analysis was performed to check correlations among the potential determinant variables. *β*-catenin was designated as the dependent variable, whereas age, BMI, BMD, estradiol, N-MID-OT, 25(OH)D, CTX, PINP, sclerostin, OPG, and RANKL were included as independent variables. In this analysis, BMD, PINP, sclerostin, and OPG were found to be independent predictors of serum *β*-catenin levels in PMOP, after adjusting for age, BMI, estradiol, N-MID-OT, 25(OH)D, CTX, and RANKL (Table 3). Scattered dots (Figures 1, 2, 3, and 4) showed the correlations of serum *β*-catenin levels with OPG, RANKL, the ratio of RANKL/OPG, and sclerostin, respectively.

## 4. Discussion 

The adult skeleton undergoes continuous remodeling through tight coupling of opposing bone-resorbing osteoclasts and bone-forming osteoblasts. The contributing elements to the function of bone homeostasis are regulated hierarchically through a series of cell signals, cross-talk, and cascades, essentially focused on members of the tumour necrosis factor superfamily RANKL and its receptors, RANK and OPG [12–14]. During normal bone remodeling, RANKL binds to the RANK transmembrane receptor on osteoclast precursors and induces differentiation and activation. OPG, also produced by BMSCs and osteoblasts, is a soluble member of the tumor necrosis factor receptor family (TNFR family), inhibits the differentiation and fusion of the osteoclastic precursor cells, and blocks the activation of mature osteoclasts [15]. When RANKL binds to RANK, osteoclast differentiation and function are enhanced [6]. Thus, targeting the RANKL/RANK/OPG system should produce potent effects on osteoclast differentiation and function [6]. Recent findings have shown that the Wnt/*β*-catenin canonical pathway in osteoblasts/stromal cells suppresses osteoclastogenesis through the upregulation of OPG expression and the downregulation of RANKL expression [16, 17].

The cross-sectional study confirms that *β*-catenin is detectable in human serum, as observed very recently by Gaudio et al. in patients with type 2 diabetes mellitus (T2DM) [18], and indicates that patients with PMOP have *β*-catenin serum levels lower than controls. *β*-catenin, a pivotal signaling molecule of the Wnt pathway, has been shown to be important in osteoblast differentiation, proliferation, and apoptosis [19]. Overexpression of *β*-catenin increased Wnt signaling activity [20]. Lower *β*-catenin levels may reflect the lower Wnt signaling activity in our PMOP cohort. Recent evidence has indicated that the Wnt/*β*-catenin pathway plays an important role in skeletal development and growth [21, 22], particularly in bone mass acquisition, remodeling, differentiation, and maintenance [23, 24]. The mechanisms are still unclear and being explored. The importance of the canonical pathway in bone biology has been emphasized by the identification of a link between bone mass and mutations in the LRP5 gene [25]. Loss-of-function mutations in LRP5 reduce the number of osteoblasts and cause osteoporosis [25, 26]. Canonical Wnts (Wnt3a) bind to the receptor complex of Frizzled and LRP5 or LRP6, inhibit GSK-3*β*, and promote the accumulation of *β*-catenin in osteoblasts [27]. The accumulated *β*-catenin translocates into the nucleus and together with TCF/LEF induces the expression of OPG to inhibit RANK-RANKL-mediated osteoclastogenesis [28]. In our study, we detected higher RANKL serum concentration and concomitantly similar OPG serum concentration at the protein levels in PMOP patients compared to controls. This suggested that there was a more seriously impaired balance between osteoblastic bone formation and osteoclastic bone resorption in patients with PMOP. In our study, we found a significant negative correlation between *β*-catenin and the ratio of RANKL/OPG. It suggested that some cross-links were present between Wnt/*β*-catenin signaling pathway and RANKL/RANK/OPG system and provided evidence that increased RANKL/OPG expression was related to reduction of Wnt/*β*-catenin signaling activity in PMOP.

We also found a significant negative correlation between *β*-catenin and sclerostin, which in fact agrees with a major contribution of sclerostin to the impairment of the Wnt/*β*-catenin signaling pathway in this setting. The pattern was unchanged in a multiple regression model. Sclerostin, an osteocyte-derived and -secreted glycoprotein, has been shown to influence the activity of Wnt signaling pathways [29]. The mechanism is not completely understood. According to the current knowledge, sclerostin antagonizes the canonical Wnt pathway by preventing the formation of the Wnt-Frizzled-LRP5 complex by competitively binding to LRP6 and LRP5, transmembrane proteins that together with Frizzled receptors mediate the actions of Wnts [4, 30–33]. Beyond that, sclerostin was found to interact with several other Wnt pathway regulatory molecules such as secreted Frizzled related protein 4 (sFRP4), casein kinase II, and TRAF2- and NCK-interacting kinase [34–37].

Both CTX and PINP significantly increased in PMOP cohort, confirming that higher bone turnover took place in this population compared to controls. However, no association between the *β*-catenin and CTX and the PINP was observed in both the PMOP group and the control group. Furthermore, we observed no correlation of serum *β*-catenin levels with age. Different from our findings, the recent report by Gaudio et al. [18] showed that there were significant correlations of serum *β*-catenin levels with age and serum sclerostin levels in T2DM patients and with age in controls. These discrepancies may be related to differences in the population characteristics. Our cohort in the study was limited to postmenopausal women with the age of 50.5–65.5 years old.

In our PMOP cohort, it is difficult to explain the negative correlation of serum *β*-catenin levels with lumbar spine BMD. For the contradictory phenomenon, the rational explanation would be that *β*-catenin is increased in a compensatory manner. Another interesting thing was that we observed a negative correlation between serum *β*-catenin and BMI. The result may be related to the role of Wnt/*β*-catenin signaling on inhibiting adipogenesis of mesenchymal precursors [38, 39].

In our control group, we found no associations of *β*-catenin with OPG and the ratio of RANKL/OPG. We hypothesized that the function communication between Wnt/*β*-catenin signaling and RANKL/RANK/OPG system was not established in postmenopausal non-osteoporosis women.

The study has some limitations. It was a cross-sectional design, and the causative nature of the associations between *β*-catenin, RANKL/OPG, and other variables cannot be established. We analyzed serum *β*-catenin, RANKL/OPG levels which may not be sensitive enough to reflect their expression in bone cells. 

## 5. Conclusions

Given the existence of multiple pathogeneses of PMOP, the recognition of the role of Wnt/*β*-catenin signaling on bone metabolism has stimulated a number of studies on the effects of this signaling on treatment of PMOP. However, the effects of Wnt signaling on bone metabolism and the involved molecular mechanisms remain unclear. According to our findings, functional communication between RANKL/RANK/OPG system and Wnt/*β*-catenin signaling pathway plays an important role in postmenopausal osteoporosis. Targeting the Wnt/*β*-catenin signaling to change the ratio of RANKL/OPG to alter bone turnover can potentially provide an approach for postmenopausal osteoporosis therapy.

## Figures and Tables

**Table 1:** The characteristics and laboratory data of patients and controls.

	PMOP group	Control group	*P*
Number	480	170	
Age (years)	58.55 ± 3.55	58.47 ± 3.52	0.813
Weight (kg)	59.43 ± 4.92	59.33 ± 4.63	0.823
Height (m)	1.54 ± 0.05	1.55 ± 0.05	0.277
BMI (kg/m^2^)	24.92 ± 1.66	24.73 ± 1.63	0.190
BMD (g/m^2^)	0.817 ± 0.073	0.997 ± 0.073	<0.001
T-score	−3.196 ± 0.599	−1.638 ± 0.542	<0.001
Estradiol (pmol/mL)	36.63 ± 15.23	38.59 ± 16.08	0.157
N-MID-OT (ng/mL)	16.49 ± 5.96	16.19 ± 5.81	0.564
25(OH)D (ng/mL)	14.68 ± 4.93	14.74 ± 4.76	0.893
CTX (ng/mL)	0.410 ± 0.086	0.323 ± 0.065	<0.001
PINP (ng/mL)	51.69 ± 9.05	46.03 ± 10.10	0.005
*β*-catenin (pg/mL)	26.26 ± 14.81	39.33 ± 5.47	<0.001
Sclerostin (pmol/L)	38.79 ± 7.43	52.86 ± 6.69	<0.001
OPG (pg/mL)	155.07 ± 91.06	157.92 ± 71.67	0.679
RANKL (pmol/L)	158.10 ± 94.53	116.03 ± 54.89	<0.001
Ratio of RANKL/OPG	1.60 ± 1.76	0.87 ± 0.45	<0.001

Data for continuous variables are presented as mean ± SD.

**Table 2:** Associations of serum *β*-catenin with other parameters in PMOP group and control group.

	PMOP group		Control group	
	*R*	*P*	*R*	*P*
Age	−0.029	0.525	−0.080	0.301
Weight	−0.038	0.400	−0.048	0.536
Height	0.038	0.402	−0.078	0.312
BMI	−0.117	0.010	0.036	0.641
BMD	−0.207	<0.001	−0.081	0.294
T-score	−0.195	<0.001	−0.085	0.272
Estradiol	0.033	0.470	−0.085	0.269
N-MID-OT	0.070	0.125	−0.047	0.542
25(OH)D	−0.031	0.493	−0.004	0.956
CTX	0.050	0.277	0.013	0.862
PINP	0.033	0.469	−0.018	0.811
Sclerostin	−0.400	<0.001	0.145	0.060
OPG	0.232	<0.001	0.112	0.145
RANKL	0.067	0.143	0.134	0.081
Ratio of RANKL/OPG	−0.128	0.005	0.032	0.674

The table shows Spearman's correlation coefficients (*R*) and associated *P* values (*P*) in PMOP group and in control group.

**Table 3:** Result for multiple regressions in PMOP group and in control group.

	PMOP group					Control group				
	*B*	SE	*β*	*t*	Sig.	*B*	SE	*β*	*t*	Sig.
Age	−0.059	0.171	−0.014	−0.348	0.728	−0.097	0.126	−0.063	−0.775	0.439
BMI	−0.312	0.364	−0.035	−0.859	0.391	0.331	0.269	0.098	1.229	0.221
BMD	−18.870	9.533	−0.093	−1.979	0.048	−3.708	5.920	−0.050	−0.626	0.532
Estradiol	−0.011	0.040	−0.011	−0.279	0.780	−0.030	0.029	−0.087	−1.014	0.312
N-MID-OT	0.173	0.103	0.070	1.682	0.093	0.010	0.083	0.011	0.126	0.900
25(OH)D	0.024	0.122	0.008	0.197	0.844	−0.004	0.099	−0.003	−0.039	0.969
CTX	−3.369	7.680	−0.020	−0.439	0.661	−7.431	8.829	−0.088	−0.842	0.401
PINP	−0.169	0.069	−0.103	−2.451	0.015	−0.025	0.047	−0.046	−0.526	0.600
Sclerostin	−0.684	0.089	−0.343	−7.721	<0.001	0.106	0.067	0.130	1.599	0.112
OPG	0.047	0.007	0.291	7.174	<0.001	0.003	0.008	0.036	0.349	0.727
RANKL	0.011	0.006	0.073	1.836	0.067	0.014	0.008	0.136	1.676	0.096
